# Prospective comparison of [^18^F]AlF-NOTA-octreotide PET/MRI to [^68^Ga]Ga-DOTATATE PET/CT in neuroendocrine tumor patients

**DOI:** 10.1186/s13550-023-01003-3

**Published:** 2023-06-01

**Authors:** Lennert Boeckxstaens, Elin Pauwels, Vincent Vandecaveye, Wies Deckers, Frederik Cleeren, Jeroen Dekervel, Timon Vandamme, Kim Serdons, Michel Koole, Guy Bormans, Annouschka Laenen, Paul M. Clement, Karen Geboes, Eric Van Cutsem, Kristiaan Nackaerts, Sigrid Stroobants, Chris Verslype, Koen Van Laere, Christophe M. Deroose

**Affiliations:** 1 Nuclear Medicine, University Hospitals Leuven and Nuclear Medicine and Molecular Imaging,, Department of Imaging and Pathology, KU Leuven, Leuven, Belgium”, Campus Gasthuisberg, Nucleaire Geneeskunde, Herestraat 49, 3000 Leuven, Belgium; 2 Radiology, Department of Imaging and Pathology, University Hospitals Leuven and Division of Translational MRI, KU Leuven, Leuven, Belgium; 3 Radiopharmaceutical Research, Department of Pharmacy and Pharmacology, KU Leuven, Leuven, Belgium; 4 Digestive Oncology, University Hospitals Leuven, Leuven, Belgium; 5 Center for Oncological Research (CORE), Integrated Personalized and Precision Oncology Network (IPPON), University of Antwerp, Antwerp, Belgium; 6 Oncology, NETwerk Antwerpen-Waasland CoE, Antwerp, Belgium; 7 Leuven Biostatistics and Statistical Bioinformatics Center, KU Leuven, Leuven, Belgium; 8 General Medical Oncology, University Hospitals Leuven, Leuven, Belgium; 9 Digestive Oncology, Department of Gastroenterology, Ghent University Hospital, Ghent, Belgium; 10 Respiratory Oncology, University Hospitals Leuven, Leuven, Belgium; 11 Nuclear Medicine, Faculty of Medicine and Health Sciences, Antwerp University Hospital and Molecular Imaging and Radiology, University of Antwerp, Wilrijk, Belgium

**Keywords:** [^18^F]AlF-NOTA-octreotide, [^68^Ga]Ga-DOTATATE, Neuroendocrine tumor, Somatostatin receptor, PET, PET/MR

## Abstract

**Background:**

Fluorine-18-labeled SSAs have the potential to become the next-generation tracer in SSTR-imaging in neuroendocrine tumor (NET) patients given their logistical advantages over the current gold standard gallium-68-labeled SSAs. In particular, [^18^F]AlF-OC has already shown excellent clinical performance. We demonstrated in our previous report from our prospective multicenter trial that [^18^F]AlF-OC PET/CT outperforms [^68^Ga]Ga-DOTA-SSA, but histological confirmation was lacking due to ethical and practical reasons. In this second arm, we therefore aimed to provide evidence that the vast majority of [^18^F]AlF-OC PET lesions are in fact true NET lesions by analyzing their MR characteristics on simultaneously acquired MRI. We had a special interest in lesions solely detected by [^18^F]AlF-OC (“incremental lesions”).

**Methods:**

Ten patients with a histologically confirmed neuroendocrine tumor (NET) and a standard-of-care [^68^Ga]Ga-DOTATATE PET/CT, performed within 3 months, were prospectively included. Patients underwent a whole-body PET/MRI (TOF, 3 T, GE Signa), 2 hours after IV injection of 4 MBq/kg [^18^F]AlF-OC. Positive PET lesions were evaluated for a corresponding lesion on MRI. The diagnostic performance of both PET tracers was evaluated by determining the detection ratio (DR) for each scan and the differential detection ratio (DDR) per patient.

**Results:**

In total, 195 unique lesions were detected: 167 with [^68^Ga]Ga-DOTATATE and 193 with [^18^F]AlF-OC. The DR for [^18^F]AlF-OC was 99.1% versus 91.4% for [^68^Ga]Ga-DOTATATE, significant for non-inferiority testing (*p* = 0.0001). Out of these 193 [^18^F]AlF-OC lesions, 96.2% were confirmed by MRI to be NET lesions. Thirty-three incremental lesions were identified by [^18^F]AlF-OC, of which 91% were confirmed by MRI and considered true positives.

**Conclusion:**

The DR of [^18^F]AlF-OC was numerically higher and non-inferior to the DR of [^68^Ga]Ga-DOTATATE. [^18^F]AlF-OC lesions and especially incremental lesions were confirmed as true positives by MRI in more than 90% of lesions. Taken together, these data further validate [^18^F]AlF-OC as a new alternative for SSTR PET in clinical practice.

*Trial registration* ClinicalTrials.gov: NCT04552847. Registered 17 September 2020, https://beta.clinicaltrials.gov/study/NCT04552847

## Background

Neuroendocrine tumors (NETs) form a heterogeneous group of tumors that have their origin in cells of the neuroendocrine system and mostly arise in the gastrointestinal and respiratory tract. The incidence and prevalence of NETs have increased significantly over the last few decades, possibly due to greater awareness of the treating physicians and pathologists and an increase in diagnostic procedures (endoscopies, diagnostic imaging) [1, 2]. Most NETs are an ideal target for molecular imaging and radionuclide therapy with somatostatin analogs (SSAs), as they are characterized by an overexpression of the somatostatin receptor (SSTR) [3]. Hence, SSTR-imaging plays a crucial and validated role in the clinical management of NETs [4]. The state of the art for SSTR-imaging nowadays is performed with positron emission tomography (PET) with [^68^Ga]Ga-DOTA-SSAs and can be executed with [^68^Ga]Ga-DOTANOC, [^68^Ga]Ga-DOTATOC and [^68^Ga]Ga-DOTATATE [5, 6]. However, as these tracers are gallium-68-labeled, they struggle with known clinical drawbacks of ^68^Ge/^68^Ga-generators such as limited availability, high associated costs, and low activity yield per elution [7]. This has spurred the development of alternative PET tracers, of which [^64^Cu]Cu-DOTATATE is a recently validated alternative in clinical practice [8–10]. Fluorine-18 is the most widely used PET radionuclide and would be an outstanding alternative, as it possesses several inherent advantages over gallium-68, such as a high production yield and more favorable half-life (109.8 min) which even allows distribution [7]. Besides that, fluorine-18 has the potential to have a higher spatial resolution because of the shorter positron range than gallium-68 [7].

Recently a promising fluorine-18 labeled SSA has been introduced for SSTR-imaging, namely [^18^F]Al-1,4,7-triazacyclononane-1,4,7-tri-acetate-octreotide; ([^18^F]AlF-NOTA-octreotide; [^18^F]AlF-OC). This tracer is manufactured using a chelator-based Al^18^F pseudo-metal method and a fast Good Manufacturing Practice-compliant process, allowing its use in clinical practice [11, 12]. Notably, [^18^F]AlF-OC has the advantage of a more favorable dosimetry, biodistribution, tracer kinetics, and lesion targeting compared with [^68^Ga]Ga-DOTA-SSA PET [13]. We recently performed a prospective, multicenter trial comparing [^18^F]AlF-OC with [^68^Ga]Ga-DOTA-SSAs and demonstrated an excellent diagnostic performance in the first PET/CT-based arm, showing superiority in 75 NET patients compared with ^68^Ga-DOTATATE/NOC [14]. In total, 4709 different tumor lesions were detected, 3454 (73.3%) with [^68^Ga]Ga-DOTATATE/NOC and 4278 (90.8%) with [^18^F]AlF-OC.

We here report the results of the second arm of this prospective trial, in which patients injected with [^18^F]AlF-OC were scanned using a PET/magnetic resonance imaging (MRI) scanner. We aimed to confirm that lesions showing [^18^F]AlF-OC uptake are genuine NET lesions using the simultaneously acquired MRI images. MRI with diffusion-weighted sequences (diffusion-weighted imaging (DWI)) is known to be highly sensitive for restricted diffusion in hypercellular malignant tumors [15], as evidenced by multiple studies reporting good sensitivity and specificity for detecting primary and metastatic NETs [16–18]We had a particular interest in MRI-based characterization of discrepant lesions with only visualization by [^18^F]AlF-OC and not by [^68^Ga]Ga-DOTA-SSA (“incremental lesions”). In the present study, we provide evidence that the majority of [^18^F]AlF-OC PET lesions are genuine NET lesions.

## Methods

### Study population

Our prospective trial consists of two arms: a PET/CT arm (part A) and a PET/MRI (part B). The results of the PET-CT arm (part A) were already presented elsewhere by Pauwels et al. [14].

**Fig. 1:**
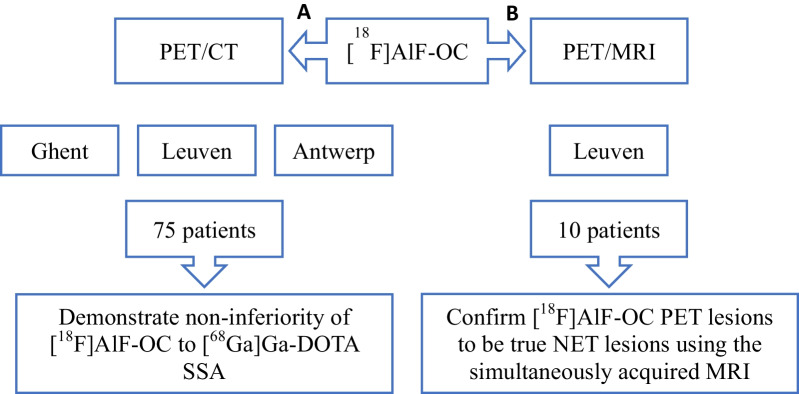
Flowchart of the study with PET/CT arm (part A) and PET/MRI arm (part B)

In the first arm (part A) of our prospective multicenter trial, 75 NET patients, aged 18 years or older, were included. In this second arm of our trial (part B), 10 other NET patients, 18 years or older, were prospectively included at University Hospitals Leuven (Fig. 1). The main inclusion criteria were: (1) histologically and/or cytologically confirmed NET of all grades of gastroenteropancreatic (GEP), pulmonary, neural crest or unknown primary origin, (2) routine clinical [^68^Ga]Ga-DOTA-SSA PET/ CT scheduled within 3 months prior or after the study scan, (3) at least one known tumor lesion with either a minimum size of 1 cm in at least one dimension on morphological imaging (CT, MRI or ultrasound), or a maximal standardized uptake value (SUV_max_) of at least 10 on [^68^Ga]Ga-DOTA-SSA PET. The main exclusion criteria were (i) previous or ongoing recurrent or chronic disease at high risk to interfere with the performance or evaluation of the trial or (ii) a contraindication for MRI imaging.

The study was performed at University Hospitals Leuven in collaboration with University Hospital Antwerp and University Hospital Ghent after approval by the Ethics Committee of all three institutes, and all subjects signed an informed consent form (ClinicalTrials.gov identifier NCT04552847).

### Tracer synthesis

[^18^F]AlF-OC was synthesized in an AllInOne® synthesis module (Trasis, Ans, Belgium) according to the procedure described by Tshibangu et al. [11], under Good Manufacturing Practice circumstances. The mean radiochemical purity of [^18^F]AlF-OC was 95.4% (range 94.3–97.6%). The mean apparent molar activity at the time of injection was 49.93 GBq/µmol (range 18.9–98.33 GBq/µmol).

[^68^Ga]Ga-DOTATATE was synthesized according to the center’s standard operating procedures, using 30 µg of good manufacturing practice-produced DOTATATE (ABX advanced biochemical compounds, Radeberg, Germany) per production.

### ***[***^***68***^***Ga]Ga-DOTATATE PET/CT acquisition***

The standard-of-care [^68^Ga]Ga-DOTATATE PET was performed according to the EANM guidelines [5] with image acquisition at 45–60 min after injection. The mean administered activity [^68^Ga]Ga-DOTATATE was 163 MBq (range 126–190 MBq).

Scans were acquired on a GE Discovery MI 4-ring PET/CT system (GE, Milwaukee, WI, USA) (3 min PET acquisition per bed position). Emission data of the GE system were iteratively reconstructed with the VPFXS algorithm (2 iterations, 34 subsets), which makes use of time-of-flight information and includes detector response modeling. PET scans were preceded by a low-dose CT for attenuation correction and anatomical information. The PET/CT system was calibrated using a uniform cylindrical phantom with gallium-68 and dose calibrator settings were adjusted so that the PET system produces images within 10% of the true SUV. To further ensure the quantitative comparison between routine and study scans, post-reconstruction filtering of PET images was done using the MIM software package, version 7.1.5 (MIM Software Inc., Cleveland, Ohio, USA) with an isotropic Gaussian smoothing kernel of 5 mm full-width half-maximum (FWHM) such that resolution properties were closely matched (based on phantom experiments, data not shown).

For both the routine and study scan, patients were asked to avoid long-acting SSA treatment, if possible, for 4 to 6 weeks prior to the scan.

### ***[***^***18***^***F]AlF-OC PET/MR acquisition***

We previously identified 2 h post-injection to be the optimal time point for [^18^F]AlF-OC PET imaging [13]. Patients underwent a whole-body PET/MRI (from mid-thigh to vertex) within an interval of a median of 119 min (range 115–157 min) after injection. [^18^F]AlF-OC was administered by a single intravenous injection of 4 MBq/kg with a maximum of 50 µg of ligand per patient. The mean injected activity and peptide mass of [^18^F]AlF-OC for all subjects were 280 MBq (range 232–340 MBq) and 10.13 μg (range 4–22 μg), respectively.

[^18^F]AlF-OC PET/MRI was performed on a 3 T GE Signa PET/MRI system (GE, Milwaukee, WI, USA). Whole-body MRI sequences were acquired simultaneously with the PET acquisitions. PET acquisition, reconstruction, and post-reconstruction filtering parameters for the GE Signa PET/MRI were identical to the GE Discovery MI 4-ring PET/CT. The MRI sequences obtained concurrently with PET were: (i) Coronal T2-weighted SSFSE, TE/TR = 80/2200, Acq matrix = 384 × 220, FOV = 440 × 440 mm, slice thickness = 5 mm, 54 slices, of the whole body of the lower abdomen and the pelvis. (ii) transversal whole-body DWI with (b values = 50, 800, TE/TR = 66/3500, Acq matrix = 96 × 128, FOV = 440 × 352 mm, slice Thickness = 5 mm, 46 slices; (iii) post-contrast transversal 3D LAVA flex sequence (water-only, fat-only, in-phase and out-phase images), GE IDEAL, dual echo, TE1/TE2/TR = 2/3/5.42, Acq matrix = 320 × 240, FOV = 420 × 336 mm, slice thickness = 5 mm, 60 slices performed during one breath-hold of the abdominal area. The post-contrast LAVA flex was acquired starting 80 s after injection of gadoterate meglumine (Dotarem®). Before the start of the abdominal part of the DWI, patients also received an injection of 20 mg Butylhyoscine bromide (Buscopan®) to minimize bowel movement. The total acquisition time was around 40–50 min.

### Image analyses

Image analysis was performed using MIM v7.1.5. Lesions with uptake greater than the physiologic uptake in the involved organs were considered candidate lesions. A lesion-by-lesion analysis of the PET images with the two different tracers, [^68^Ga]Ga-DOTATATE and [^18^F]AlF-OC, was performed in consensus by two experienced readers, blinded for patient data but unblinded for the radiopharmaceutical that was used as by design the hybrid partner (CT or MRI) would reveal the tracer. For each candidate lesion, a Likert score was given on a scale of 1 to 5, which correlated with the probability of the candidate lesion to be a NET lesion. A lesion with a Likert score of 1 was estimated not to be a NET lesion, with SSTR expression caused by a benign or physiological etiology (“benign”), while a lesion with a Likert score of 5 was considered a NET lesion (“malignant”). Scores of 2 and 4 represented probable benign and probable malignant lesions, respectively. A Likert score of 3 was equivocal. All candidate lesions with a Likert score of 3 or higher were suspicious to be NET lesions and were considered positive lesions and included in the lesion analysis. For the union of positive lesions identified with both tracers, the other scan was investigated for corresponding lesions. If there was a difference of more than 2 points in the Likert score between the two corresponding lesions or when there was no corresponding lesion detected on the other scan, the lesion was defined as an incremental lesion.

Region of interest (ROIs) were drawn manually on PET images to obtain SUVs. For each lesion, the SUV_max_ was measured and the tumor-to-background ratio (TBR) was calculated by dividing the SUV_max_ of that lesion by the SUV_mean_ of relevant background tissue (liver for liver lesions, bone for bone lesions, and gluteal muscle for all other lesions).

ROIs for measuring the SUV_max_ and SUV_mean_ were drawn in healthy liver, bone, and muscle tissue for measuring normal background uptake. A sphere of 2 cm was drawn in the right liver lobe, two ROIs were drawn in the vertebral body of two consecutive vertebrae of the lower dorsal spine, and an ROI was drawn in the gluteal muscles on both sides for five consecutive slices for measuring normal uptake in healthy liver, bone, and muscle tissue, respectively.

All PET lesions were investigated for a corresponding lesion on MRI images by an experienced radiologist, unblinded for the tracer used (see above). MRI score sheets were checked for corresponding lesions for the union of candidate lesions (Likert score 1–5) identified on both PET scans. A NET lesion was defined by hyperintensity on b800 DWI MRI, not attributable to T2 shine-through or anatomical structure. Lymph nodes were considered to be NET lesions if the b800 DWI signal intensity was equal to or higher than the solid component of the primary tumor and/or higher relative to surrounding lymph nodes, and irrespective of nodal size [19].

In candidate lesions with a Likert score ≤ 2, MRI score sheets were checked to confirm a benign or physiological etiology. If MRI could confirm a NET lesion in a positive PET lesion (with a Likert score ≥ 3), the lesion was defined as an MRI-confirmed lesion. When MRI showed another etiology causing the elevated tracer uptake this was noted.

The detection ratio (DR) was determined for each scan, i.e., the fraction of positive lesions detected on that scan, using the union of positive lesions detected by both tracers in a patient as the reference. Finally, the differential detection ratio (DDR), which is the difference in DR between [^18^F]AlF-OC and [^68^Ga]Ga-DOTATATE, was calculated for each patient. The DR and the DDR were also determined for the MRI-confirmed lesions.

### Statistical analyses

Statistical analyses were performed using the SAS software (v9.4 of the SAS System for Windows). A one-sample t test was used to test the difference in DR between both tracers at the patient level. The difference in DR, background uptake, and lesion uptake at lesion level or organ level (mean SUV_max_ or mean TBR per lesion or per organ) was compared using linear mixed models including random intercepts to account for data clustering. The difference in background uptake and lesion uptake at the patient level (mean SUV_max_ or mean TBR per patient) was analyzed using linear models. The normality of the model residuals was checked graphically. When relevant, 95% confidence intervals (CI) were computed. Two-sided *P* values less than 0.05 were considered significant. The study was designed to demonstrate non-inferiority in arm A, with a threshold of -15% for the DDR [14]. As the results in arm A showed both significance for non-inferiority and superiority, both tests were performed in arm B.

## Results

### ***Patient selection and [***^***18***^***F]AlF-OC administration***

Ten patients (7 males, 3 females; aged 38–71) were prospectively enrolled in the study. Their clinical and tumor characteristics are summarized in Tables 1 and 2. The median time between standard-of-care [^68^Ga]Ga-DOTATATE and [^18^F]AlF-OC PET was 9.5 days (range -8 to + 35 days). No therapeutic changes occurred between the scans. The median time interval between the last treatment and the PET/CT scan was 40 ± 9 days (range 20–54) and 43 ± 20 days (range 26–89) for the PET/MR. The TNM stage was determined on the standard-of-care [^68^Ga]Ga-DOTATATE PET/CT, according to the 8th edition of the UICC.

**Table 1:** Patient and clinical characteristics (n = 10)

Characteristic	Number (%) of patients or Median (range)
Age (y)	60 (38–71)
Sex	
Male	6 (60%)
Female	4 (40%)
Primary tumor	
Pancreas	4 (40%)
Intestine	2 (20%)
Lung	2 (20%)
CUP	2 20%)
Tumor grade	
G1	2 (20%)
G1/G2 (i.e., Ki-67 < 5%)	2 (20%)
G2	6 (60%)
Ki-67 (%)	5 (1–18)
Ongoing therapy at time of scan	
SSA only	6 (60%)
SSA and everolimus	2 (20%)
None	2 (20%)
Interval [^18^F]AlF-OC and [^68^Ga]Ga-DOTATATE scan (days)	9.5 (− 8 to + 35)
TNM stage	
T (0/1/2/3/4)	T (8/0/2/0)
N (0/1/2/3)	N (4/5/0/1)
M (0/M1a/M1b/M1c)	M (1/1/2/6)

**Table 2:** Individual patient characteristics

Patient	Age	Sex	Primary tumor	TNM	Grade	Ki-67 index	Ongoing therapies	First scan	Scan interval (days)	Time between scan and therapy (days)	
										F-18	Ga-68
1	64	M	Pancreas	T0N1M1c	G2	2–10%	SSA, everolimus	[^68^Ga]Ga-DOTATATE	16	45	61
2	54	V	Pancreas	T2N0M1c	G2	18%	SSA	[^68^Ga]Ga-DOTATATE	22	46	68
3	61	M	Pancreas	T0N1M1c	G2	10%	/	[^68^Ga]Ga-DOTATATE	14	/	/
4	71	V	Pancreas	T2N0M0	G1	2%	/	[^68^Ga]Ga-DOTATATE	13	/	/
5	60	V	Small Intestine	T0N1M1c	G2	8%	SSA	[^68^Ga]Ga-DOTATATE	4	41	45
6	60	M	Small Intestine	T0N1M1c	G1	1%	SSA	[^18^F]AlF-OC	1	40	39
7	48	M	Lung	T0N3M1c	G1/G2	< 5%	SSA	[^18^F]AlF-OC	8	35	27
8	38	M	Lung	T0N0M1b	G2	5%	SSA	[^68^Ga]Ga-DOTATATE	35	54	89
9	57	V	CUP	T0N0M1b	G2	7%	SSA, everolimus	[^68^Ga]Ga-DOTATATE	6	20	26
10	65	M	CUP	T0N1M1a	G1/G2	< 5%	SSA	[^68^Ga]Ga-DOTATATE	4	37	41

### PET Lesion analysis

In total 200 unique suspected PET lesions were detected; 198 with [^18^F]AlF-OC compared to 172 with [^68^Ga]Ga-DOTATATE. Five lesions with a Likert score ≤ 2 were excluded from the final lesion analysis, resulting in 195 unique PET-positive lesions (Likert score ≥ 3) of which 193 with [^18^F]AlF-OC and 167 with [^68^Ga]Ga-DOTATATE.

**Fig. 2:**
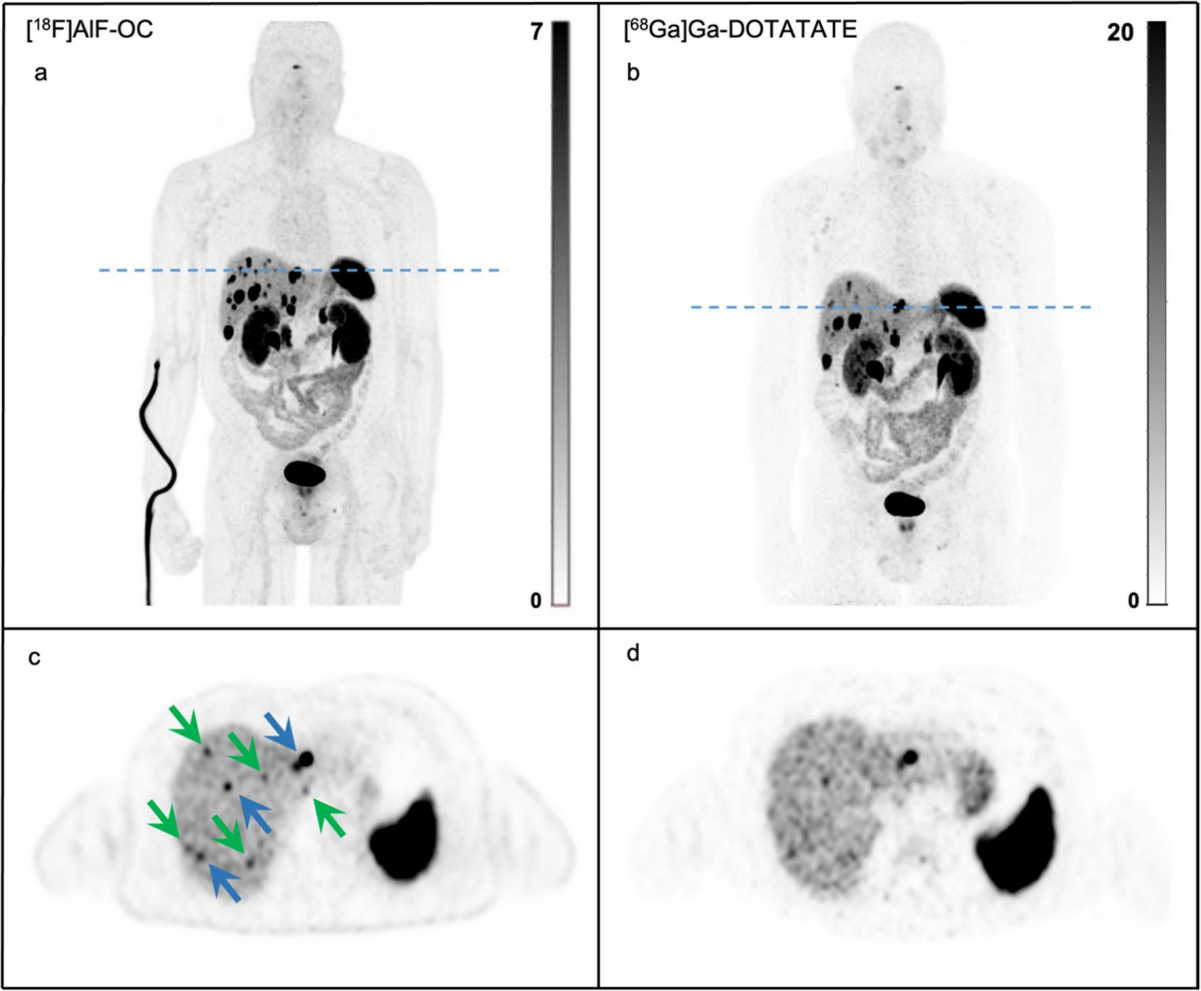
**A/C** [^18^F]AlF-OC and **B/D** [^68^Ga]Ga-DOTATATE images (from top to bottom: maximum-intensity projection PET and transverse PET) of a 60-year-old male patient with a history of resection of small intestinal NET, currently with liver metastases. Multiple liver lesions were called negative by [^68^Ga]Ga-DOTATATE and are shown by green arrows. Blue arrows indicate concordant lesions

**Fig. 3:**
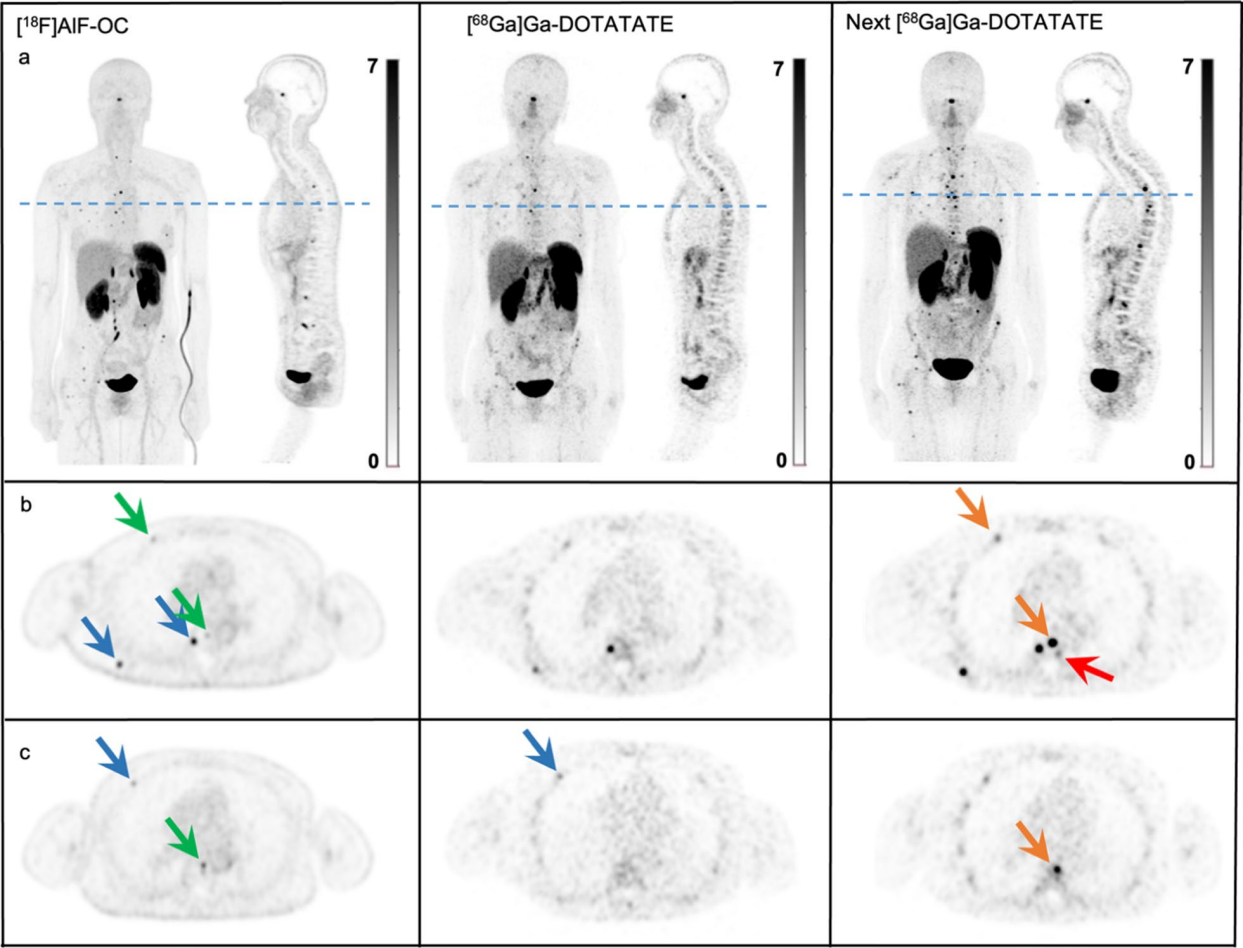
**a** Maximum-intensity projections and sagittal images of [^18^F]AlF-OC, [^68^Ga]Ga-DOTATATE PET and of [^68^Ga]Ga-DOTATATE PET 4 months after aforementioned scans of a 55-year-old female patient with a pancreatic NET with multiple bone metastases. **b** Transverse PET images with two concordant bone lesions on [^18^F]AlF-OC and [^68^Ga]Ga-DOTATATE (blue arrows) and two incremental bone lesions with [^18^F]AlF-OC (green arrows). Both incremental bone lesions were visible on the next clinical follow-up [^68^Ga]Ga-DOTATATE scan 4 months later (orange arrow), as well as a new bone lesion (red arrow). **c** Transverse PET images showing one concordant bone lesion on both scans (blue arrow) and one incremental bone lesion with [^18^F]AlF-OC (green arrow). The incremental bone lesion had no corresponding lesion on MRI, but the next clinical follow-up [^68^Ga]Ga-DOTATATE scan 4 months later (orange arrow) showed obvious tracer uptake

Most [^18^F]AlF-OC lesions were detected in bone (77 lesions in 7 patients), followed by liver (64 lesions in 7 patients), lung (20 lesions in 1 patient), lymph nodes (18 lesions in 6 patients) and other organs (16 lesions in 4 patients). Other organ localizations included the peritoneum, pancreas, heart, uterus, and ovarium. Both [^18^F]AlF-OC and [^68^Ga]Ga-DOTATATE revealed incremental lesions (i.e., a lesion seen by one PET tracer but not by the other). [^18^F]AlF-OC detected 33 incremental lesions (16 in the liver, 15 in the bone, 1 in the lung, and 1 in the peritoneum), while [^68^Ga]Ga-DOTATATE identified 3 incremental lesions: two liver lesions and one lymph node. [^18^F]AlF-OC revealed 13 incremental lesions in the liver in a single patient (patient 6; Fig. 2) and 14 incremental bone lesions in another patient (patient 3; Fig. 3).

A trend toward a higher DR with [^18^F]AlF-OC than with [^68^Ga]Ga-DOTATATE (99.1% vs 91.4%) was observed, resulting in a mean DDR of 7.7% (95% CI − 0.4–15.8) in favor of [^18^F]AlF-OC (*p* = 0.059). While the mean DR of [^18^F]AlF-OC was not high enough to be significantly superior to [^68^Ga]Ga-DOTATATE, it was highly significant for non-inferiority testing (*p* = 0.0001). Table 3 shows the mean DR of the lesions and the most prevalent organs. The mean DR per organ was always significantly non-inferior with [^18^F]AlF-OC compared to [^68^Ga]Ga-DOTATATE, but never significantly superior.

**Table 3:** Comparison between the mean detection ratio of all PET lesions with ^68^Ga-DOTATATE (DR_Ga_) and [^18^F]AlF-OC (DR_F_) and mean differential detection ratio (DDR) with 95% confidence interval (95% CI) for the most relevant organs

Organ	mean DR_Ga_ (%)	mean DR_F_ (%)	Mean DDR (%)	95% CI (%)	*P*_*superiority*_	P_non-inferiority_
Liver	85.4	97.6	12.2	− 4.9–29.4	0.13	0.0081
Bone	96.1	100	3.9	− 3.2–11.1	0.23	0.0006
Lymph nodes	100	100	0	/	/	/
Lung	95	100	5	/	/	/
Other	96.7	100	3.3	− 5.2–11.9	0.37	0.0027
All	91.4	99.1	7.7	− 0.4–15.8	0.059	0.0001

### MRI correlation

MRI showed a corresponding lesion in 185 out of 193 [^18^F]AlF-OC positive lesions (95.8%) and in 161 out of 167 [^68^Ga]Ga-DOTATATE positive lesions (96.4%). In 4 mutual PET lesions, physiological [^18^F]AlF-OC (residual) uptake was considered in the ureter, a blood vessel, a nerve root, and the bowel wall. Only 4 [^18^F]AlF-OC lesions and 2 [^68^Ga]Ga-DOTATATE lesions did not show a corresponding suspect lesion on MRI, consisting of two bone lesions (mutually observed with both PET tracers) and one other bone lesion and one liver lesion observed with [^18^F]AlF-OC.

Out of the 185 [^18^F]AlF-OC positive lesions, 178 (96.2%) were NET lesions on MRI and were considered MRI-confirmed lesions. For the other 7 [^18^F]AlF-OC lesions, MRI revealed [^18^F]AlF-OC uptake to be attributed to a benign etiology with known SSTR expression, namely three myofibromas in the uterus, three schwannomas in bone lesions and a hemangioma in the dorsal spine. MRI revealed 155 NET lesions out of the 165 (96.2%) [^68^Ga]Ga-DOTATATE positive lesions and benign [^68^Ga]Ga-DOTATATE uptake was observed in the same lesions as with [^18^F]AlF-OC, except one schwannoma.

The MRI-confirmed NET lesions were detected in the bone (68 lesions in 7 patients), liver (63 lesions in 7 patients), lung (20 lesions in 1 patient), lymph nodes (18 lesions in 6 patients), peritoneum (6 lesions in 3 patients), pancreas (3 lesions in 2 patients), heart (1 lesion in 1 patient) and in the brain (1 lesion in 1 patient). The mean DR for the MRI-confirmed NET lesions was 98.9% with [^18^F]AlF-OC and 91.9% with [^68^Ga]Ga-DOTATATE. The resulting mean DDR of the MRI-confirmed NET lesions was 7.1% in favor of [^18^F]AlF-OC, again significantly non-inferior (*p* = 0.014), but not meeting the criteria for superiority (*p* = 0.0595) (Table 4).

**Table 4:** Comparison between the mean detection ratio of all MRI-confirmed NET lesions with [^68^Ga]Ga-DOTATATE (DR_Ga_) and [^18^F]AlF-OC (DR_F_) and mean differential detection ratio (DDR) with 95% confidence interval (95% CI) for the most relevant organs

Organ	Mean DR_Ga_ (%)	Mean DR_F_ (%)	Mean DDR (%)	95% CI (%)	*P*_*superiority*_	P_non-inferiority_
Liver	88.2	97.6	9.4	− 7.9–26.7	0.23	0.014
Bone	96.1	100	3.9	− 2.7–10.6	0.20	0.0004
Lymph nodes	100	100	0	/	/	/
Lung	95	100	5	/	/	/
Other	95.8	100	4.2	− 6.5–14.9	0.36	0.0058
All	91.9	98.9	7.1	− 1.5–15.6	0.096	0.0003

In total, 36 incremental lesions were observed with [^18^F]AlF-OC and [^68^Ga]Ga-DOTATATE, of which 33 with [^18^F]AlF-OC. MRI could confirm these incremental lesions to be genuine NET lesions in 33 lesions, of which 30 [^18^F]AlF-OC lesions (91%) and all 3 [^68^Ga]Ga-DOTATATE lesions (100%). Two [^18^F]AlF-OC lesions showed no corresponding lesion on MRI and one [^18^F]AlF-OC lesion in the dorsal spine showed a schwannoma on MRI. In total, 91% of the incremental lesions with [^18^F]AlF-OC were confirmed to be NET lesions by MRI.

Both PET tracers had 5 mutual candidate lesions with a Likert score ≤ 2, deemed to be caused by physiological or benign etiology. MRI could confirm these lesions to be two meningiomas in the brain and two splenosis lesions in the peritoneum. One mutually visualized lesion in the dorsal spine did not have a corresponding lesion on MRI and was probably caused by degenerative changes.

### Lesion uptake

At the lesion level, the SUV_max_ of the MRI-confirmed lesions showed a trend toward higher values in all organs with [^68^Ga]Ga-DOTATATE compared with [^18^F]AlF-OC (mean difference 13.3, *p* = 0.053). Background uptake was significantly lower with [^18^F]AlF-OC for healthy liver (*p* = 0.019), bone (*p* =  < 0.0001), and muscle (*p* = 0.0008). The numerically higher SUV_max_ of the lesions with [^68^Ga]Ga-DOTATATE and significantly lower SUV_mean_ in the normal organs with [^18^F]AlF-OC resulted in a similar tumor-to-background ratio (TBR) (Tables 5, 6).

**Table 5:** Mean SUV_max_ and tumor-to-background ratio (TBR) with [^68^Ga]Ga-DOTATATE (SUV_max___Ga_; TBR_Ga_) and with [^18^F]AlF-OC (SUV_max__F; TBR_F_) at patient level for all concordant, quantifiable lesions (n = 153)

Organ	Mean SUV_max_Ga_	Mean SUV_max_F_	*P*	Mean TBR_Ga_	Mean TBR_F_	*P*
Liver	30.9 ± 24.0	16.6 ± 12.5	0.086	4.8 ± 4.3	5.1 ± 4.0	0.41
Bone	12.2 ± 30	6.6 ± 10.3	0.24	7.4 ± 23.7	8.4 ± 16.4	0.83
Lymph nodes	23 ± 14.8	16.5 ± 18.6	0.51	37.8 ± 26.4	40.1 ± 43.5	0.85
Lung	18.3 ± 16	7.3 ± 5.8	/	28.1 ± 24.6	20.1 ± 16.2	/
Other	27.6 ± 20.6	14.6 ± 7.8	0.031	44.1 ± 31.8	36.6 ± 22.1	0.37
All	21.1 ± 25.7	11.5 ± 12.6	0.053	15.1 ± 24.9	14.4 ± 23.1	0.81

No *p* value for the lung lesions (all observed in a single patient) was provided as our statistical analysis technique did not allow to compare observations coming from a single individual

**Table 6:** Mean SUV_mean_ in healthy organs with [^68^Ga]Ga-DOTATATE (SUV_mean Ga_) and with [^18^F]AlF-OC (SUV_mean___F_)

Organ	Mean SUV_mean_Ga_	Mean SUV_mean_F_	*P*
Liver	6.7 ± 2.9	4.3 ± 1.8	0.019
Bone	1.9 ± 0.6	0.7 ± 0.2	< 0.0001
Muscle	0.6 ± 0.1	0.4 ± 0.05	0.0008

## Discussion

**Fig. 4:**
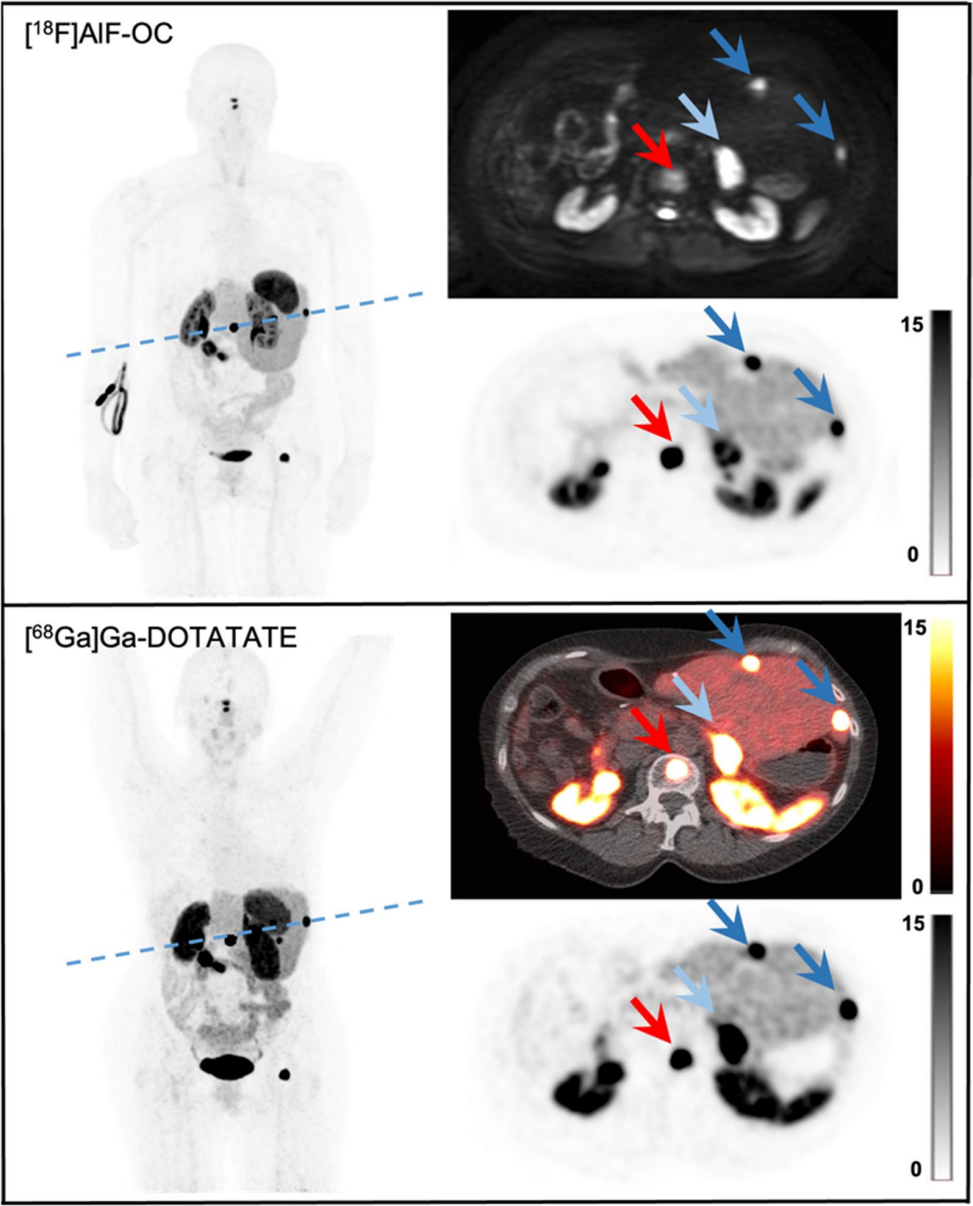
[^18^F]AlF-OC (from left to right, clockwise: maximum-intensity projection PET, diffusion-weighted imaging (DWI b = 800) MRI and tilted transvere PET) and [^68^Ga]Ga-DOTATATE images (from left to right, counterclockwise: maximum-intensity projection PET, transversal PET, and fused PET-CT images) of a 65-year-old male patient with a NET with brain, bone, liver, and lymph node metastases. The transverse slices were tilted to illustrate more lesions in 1 slice. All [^18^F]AlF-OC have a corresponding lesion on DWI MRI. The light blue arrow indicates the primary lesion in the tail of the pancreas, the blue arrows indicate two liver metastases, and the red arrow indicates a bone metastasis. All lesions were also visible with [^68^Ga]Ga-DOTATATE (i.e., concordant lesions)

**Fig. 5:**
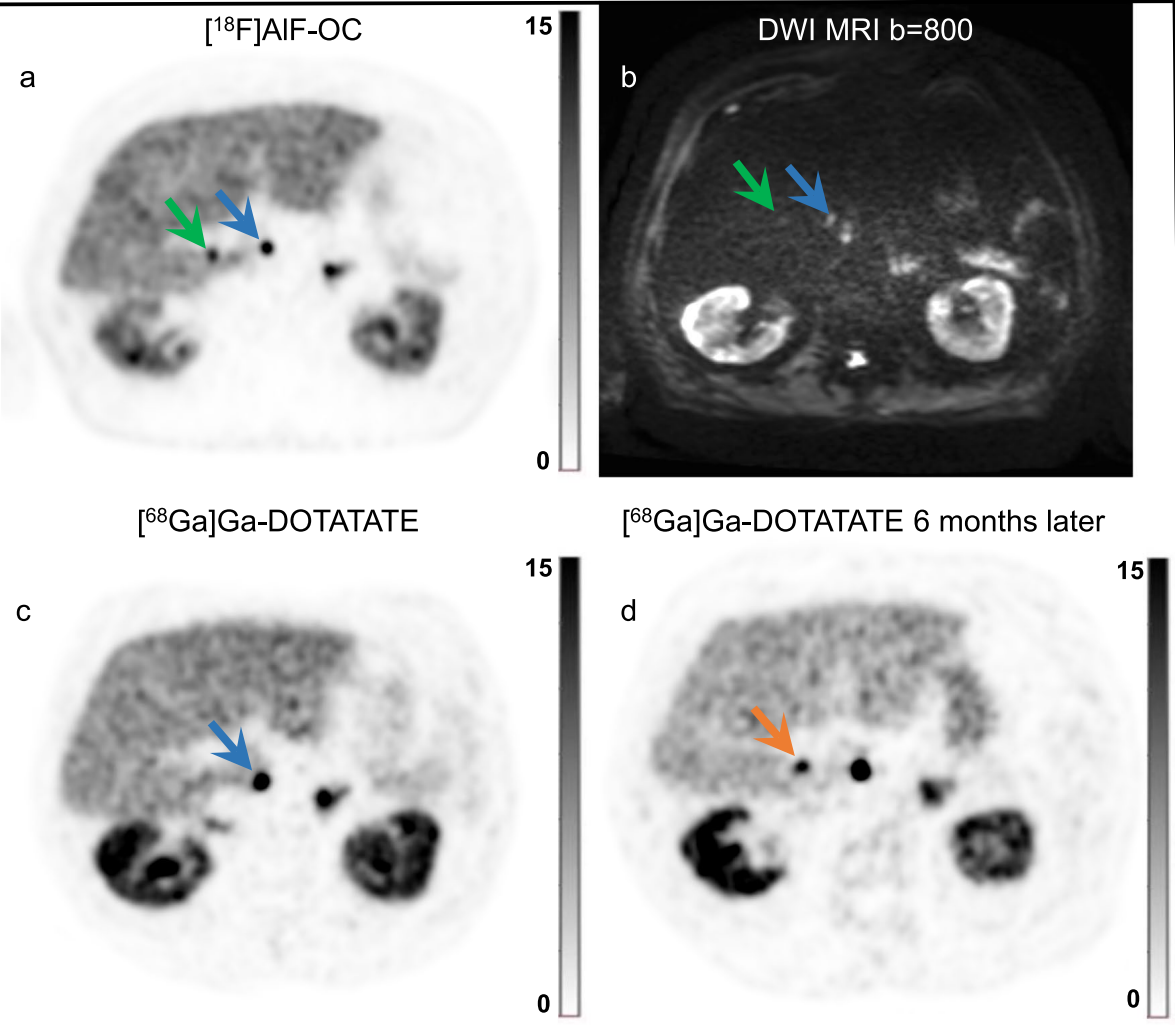
Transverse PET images with **A** [^18^F]AlF-OC and **C** [^68^Ga]Ga-DOTATATE showing a concordant liver lesion (blue arrow) and an incremental liver lesion with [^18^F]AlF-OC (green arrow). **B** DWI MRI shows a corresponding lesion for both liver lesions and therefore confirms the incremental lesion. **D** [^68^Ga]Ga-DOTATATE PET 6 months later also shows the incremental liver lesion (orange arrow)

Fluorine-18-labeled SSAs have the potential to become the next-generation tracer in SSTR-imaging in NET patients given their logistical advantages over the current gold standard gallium-68-labeled SSAs. In particular, [^18^F]AlF-OC has already shown excellent clinical performance. Indeed, in our recent prospective multicenter trial, we showed that PET/CT with [^18^F]AlF-OC was superior compared to [^68^Ga]Ga-DOTA-SSA; however, due to ethical and practical reasons histological confirmation was not available. In the present study, we therefore aimed to confirm the hypothesis that most [^18^F]AlF-OC PET lesions are true NET lesions by using their MR characteristics on simultaneously acquired MRI. Using this approach, we observed that 95.8% of the [^18^F]AlF-OC lesions had a corresponding anatomical lesion on MRI, of which 96.2% were in fact true NET lesions (Fig. 4). Thirty-three incremental lesions were detected with [^18^F]AlF-OC over [^68^Ga]Ga-DOTATATE, of which 30 lesions (91%) were confirmed true NET lesions on MRI (Fig. 5). These findings further validate [^18^F]AlF-OC as a clinically highly performant option for a standard-of-care SSTR PET.

These findings are in line with a recent study by Hou et al., where 28 incremental lesions were observed with [^18^F]AlF-OC in 20 NET patients. Of interest, all lesions were confirmed to be true NET lesions by follow-up CT and/or MRI [20]. In our study, two incremental lesions, one in liver and one in bone, were initially not confirmed by MRI. This, however, does not fully rule out that these lesions can be true NET lesions, as the overall sensitivity of SSTR PET/CT can be higher than for DWI MRI imaging [21]. This indeed applied for the incremental bone lesion, as the next clinical follow-up [^68^Ga]Ga-DOTATATE PET/CT 4 months later clearly showed elevated tracer uptake in the bone lesion (Fig. 3). The incremental liver lesion without MRI correlate has so far not been observed on clinical [^68^Ga]Ga-DOTATATE PET/CT during further follow-up, and thus might be a false positive. This can possibly be explained by benign focal radiotracer uptake, in line with previous studies reporting DWI MRI to be superior in detecting liver metastases [18, 22]. One incremental lesion turned out to be a schwannoma in the dorsal spine.

Approximately 80% of NET patients will develop metastases in the liver during the course of their disease, making the liver the main organ for distant metastases [23]. As the liver tumor burden is a crucial factor in the decision to offer curative locoregional treatment (ranging from ablation to even liver transplantation), it is of extreme importance to have access to a sensitive and accurate diagnostic technique to estimate liver involvement. Some studies report a higher detection rate for liver metastases with MRI than with [^68^Ga]Ga-DOTA-SSA PET/CT, suggesting that combining MRI with SSTR-PET may represent a promising diagnostic improvement [23]. MRI mainly outperforms [^68^Ga]Ga-DOTA-SSA PET/CT in detecting small liver lesions, especially with the use of dynamic IV gadolinium contrast-enhanced examination or DWI [17, 22]. In one patient in our cohort, MRI indeed detected more liver lesions than [^18^F]AlF-OC PET and [^68^Ga]Ga-DOTATATE PET, which were all small lesions. PET/MRI is predominantly superior due to the MRI component and its superiority to CT for detecting metastases to the liver, pancreas, bone, and brain [24–26]. Conversely, PET/CT is advised for the detection of lung lesions. The best hybrid partner (CT or MRI) for SSTR-imaging therefore depends on the presentation of the disease.

In the per-organ analysis of the first arm of our study, we demonstrated that [^18^F]AlF-OC outperformed [^68^Ga]Ga-DOTATATE/NOC in the liver with a mean DDR of 33% [14]. These data indicate that SSTR-imaging with [^18^F]AlF-OC PET/MRI seems promising when liver involvement is suspected and especially when amenable to locoregional treatment.

In our study, MRI also revealed seven PET lesions (3.8%) caused by another SSTR-positive entity, like a schwannoma, a fibromyoma, or a hemangioma, and confirmed benign SSTR-positive entities in four occasions, namely two meningiomas and two splenosis lesions. These benign etiologies are known pitfalls in SSR-imaging [27, 28].

We also investigated the diagnostic performance in terms of the detection rate of [^18^F]AlF-OC PET/MRI compared with [^68^Ga]Ga-DOTATATE PET/CT. The mean DDR of the PET lesions and the MRI-confirmed lesions was 7.7% and 7.1%, respectively, both in favor of [^18^F]AlF-OC and meeting the significance criteria for non-inferiority testing. This is in line with the findings of the first arm of our multicenter study where we observed a mean DDR of 15.8%, demonstrating the superiority of [^18^F]AlF-OC compared with [^68^Ga]Ga-DOTATATE/NOC [14]. Unlike the first arm of our multicenter trial, the current study showed non-inferiority, but not superiority, of [^18^F]AlF-OC, even with the current small sample size.

This study is an elegant example on how hybrid simultaneous PET/MRI can help in the development of new PET tracers with added value over PET/CT. In particular in bone and small lymph nodes, CT will show less lesions and will not allow to validate the uptake of the novel tracer. PET/MRI has previously been used in PET tracer development for first-in-man studies, where the reduced radiation dose is a strong advantage, but has also been done in a range of tumor types, including prostate cancer and melanoma [29–31]**.**

Lesion uptake quantified by SUV_max_ showed a trend toward a higher SUV_max_ with [^68^Ga]Ga-DOTATATE compared with [^18^F]AlF-OC for all organs. These results are in line with our previous findings but differ from those of Hou et al. who observed higher SUV_max_ with [18F]AlF-OC [20]. Lower uptake might hamper the detection of lesions. However, the TBR and therefore the image contrast which drives lesion detection was similar of even higher with [^18^F]AlF-OC, because of the significantly lower background uptake in all organs with [^18^F]AlF-OC compared to [^68^Ga]Ga-DOTATATE. This needs further study in particular organs, e.g., the pancreas for the detection of small pancreatic NETs. This study has several limitations. The first limitation is the sample size and therefore the limited statistical power, which is probably the main reason why this part of the study could not significantly confirm the superiority of [^18^F]AlF-OC compared with [^68^Ga]Ga-DOTATATE, in contrast with arm A with a study population of 75 patients. Because of the small sample size, the patient population does not include a large sampling of NETs with different origins and grades. We do not provide data in patients with Ki-67 > 20% and in NEC. However, we think the data of our trial need to be seen in its totality, including the data from the 75 patients scanned on PET/CT by Pauwels et al. [14]. The second limitation is potential bias during image reads as both readers were unblinded for the tracer, because the nature of the tracer could easily be deduced from the hybrid morphological modality. The analysis was also performed side-by-side PET-wise to immediately correlate for a corresponding lesion with the other tracer, which potentially leads to less incremental lesions, as lesions with faint uptake on one tracer can sometimes still be identified on the other modality. Thirdly, in an ideal setting two PET/MR scans would have been performed to allow a head-to-head comparison but this was practically (no reimbursement of PET/MR) and ethically not possible to perform a total of 3 PET scans (1 routine PET/CT and 2 study PET/MRI). Furthermore, we were mainly interested in the MR characterization of the [^18^F]AlF-OC lesions. Another limitation is that the radiologist was not blinded for the data of the PET, and therefore correlation was easier but not independent. Due to practical reasons, it was also not always possible to organize the PET/MR study scan within a few days from the [^68^Ga]Ga-DOTATATE PET/CT scan. However, as all patients had a G1 or G2 tumor grade it is very unlikely to develop new lesions in this timeframe in this disease type. Finally, there is an important difference in imaging parameters between the two PET tracers which at least in part contributes to the better detection rate of [^18^F]AlF-OC. The higher administered activity and longer time between tracer administration and imaging with [^18^F]AlF-OC compared with [^68^Ga]Ga-DOTATATE is an inherent advantage of fluorine-18-labeled tracers that should be exploited. In this sense, our study compares two real-life clinical strategies rather than comparing two radiopharmaceuticals by themselves.

## Conclusion

[^18^F]AlF-OC performed non-inferior to [^68^Ga]Ga-DOTATATE and incremental lesions were confirmed by MRI in more than 90% of lesions as true positives. This supports the findings of the recently reported other arm of our prospective multicenter trial and further validates [^18^F]AlF-OC as a promising novel option for clinical practice SSTR PET.

## Data Availability

The datasets generated and/or analyzed during the current study are not publicly available and are not approved by the Ethics Committee at UZ/KU Leuven due to patient's confidentiality issues, but are available from the corresponding author upon reasonable request.
